# Assembly of Bacterial Genomes from the Metagenomes of Three Lichen Species

**DOI:** 10.1128/MRA.00622-20

**Published:** 2020-09-17

**Authors:** Wisnu Adi Wicaksono, Tomislav Cernava, Martin Grube, Gabriele Berg

**Affiliations:** a Institute of Environmental Biotechnology, Graz University of Technology, Graz, Austria; b Institute of Biology, University of Graz, Graz, Austria; Georgia Institute of Technology

## Abstract

Bacteria have recently emerged as important constituents of lichen holobionts. Here, 29 bacterial metagenome-assembled genome (MAG) sequences were reconstructed from lichen metagenomes and taxonomically classified in four phyla. These results provide a pivotal resource for further exploration of the ecological roles played by bacterial symbionts in lichen holobionts.

## ANNOUNCEMENT

Lichenized fungi are known as symbiotic associations of a mycobiont (fungus) and a photobiont (green alga and/or cyanobacterium). Recently, evidence was found for the presence of bacterial communities that play important roles in this symbiotic system (1–4). Surprisingly little is known about the genomes of dominant but so far uncultured bacteria in these miniature ecosystems. Here, we present bacterial metagenome-assembled genomes (MAGs) that were reconstructed from three lichen metagenome samples, i.e., Lobaria pulmonaria (L.) Hoffm., Cladonia furcata (Huds.) Schrad., and Peltigera polydactylon (Neck.) Hoffm. (3, 5). The selected lichens represent variants of symbiotic associations of the mycobiont with one or two types of photobionts. The lung lichen L. pulmonaria includes a green alga (*Dictyochloropsis reticulata*) and a cyanobacterium (*Nostoc* sp.) as photobionts (6), and P. polydactylon includes a cyanobacterium (*Nostoc* sp.) (7). In contrast, the genus *Cladonia* includes only a green alga (*Asterochloris* sp.) (8).

Lichen samples were obtained from three locations in Austria (3, 5). Metagenomic DNA was extracted using the MO BIO PowerSoil DNA isolation kit. The metagenomic DNA was sequenced by GATC Biotech (Konstanz, Germany) after libraries were prepared with the Illumina TruSeq DNA library kit. The Illumina HiSeq 2000 (L. pulmonaria) and HiSeq 2500 (C. furcata and P. polydactylon) instruments were used for paired-end 100- or 150-bp sequencing, resulting in >35 million reads per metagenome. Community-level assessments of bacterial functioning using these metagenome data sets were reported elsewhere (3, 5).

Default parameters were used for all software unless otherwise noted. Illumina adaptor removal and initial filtering of low-quality reads (Phred scores of <20) were performed using Trimmomatic v0.39 and VSEARCH v2.14.2 (9, 10). Metagenome data sets were then *de novo* assembled using metaSPAdes v3.14.0 (11). Totals of 103,819, 135,511, and 68,049 contigs with a length of >1 kb were generated from the *Cladonia*, *Lobaria*, and *Peltigera* metagenome data sets, respectively. The generated contigs were binned using MaxBin2 v2.2.7, MetaBAT2 v2.12.1, and CONCOCT v1.1.0 (12–14) and were further dereplicated and aggregated into MAGs using DAS Tool v1.1.1 (15). The completeness and the percentage of contaminations in the MAGs were estimated using CheckM v1.0.13 (16). The quality of the MAGs was classified according to the Minimum Information about a Metagenome-Assembled Genome (MIMAG) standards (17). The Bin Annotation Tool v4.6 was used to obtain the taxonomic classification for each MAG (18).

Twenty-nine MAGs with contamination of <10% were recovered. Among them, 7, 17, and 5 MAGs originated from the *Cladonia*, *Lobaria*, and *Peltigera* metagenomes, respectively. The MAGs were assigned to *Proteobacteria* (20 MAGs), *Acidobacteria* (3 MAGs), *Bacteroidetes* (3 MAGs), and *Verrumicrobia* (1 MAG) (Table 1). One MAG each was classified in the candidate phylum “*Candidatus* Parcubacteria” and the superphylum *Terrabacteria*. We recovered 8 high-quality, 18 medium-quality, and 3 low-quality draft MAGs. The estimated completeness of the MAGs ranged from 26.9 to 98.9%, and genome sizes ranged from 492,776 to 5,800,883 bp. To the best of our knowledge, our data present the first MAGs recovered from lichen metagenomes. They provide an extended basis for further exploration of the symbiotic function of lichen-associated bacteria that will be conducted in follow-up studies.

**TABLE 1:** Detailed taxonomic classification, assembly characteristics, and GenBank accession numbers for bacterial MAGs

MAG alias	Taxonomic classification	Completeness (%)	Contamination (%)	Genome size (bp)	GC content (%)	MIMAG classification	GenBank accession no.
Lichen_MAGs_cladonia1	*Caulobacter* sp. strain S45	95.2	2.5	3,258,825	68.8	High	CAHJWH000000000
Lichen_MAGs_cladonia2	*Sphingomonas*	96.9	0.4	3,049,150	66.2	High	CAHJWJ000000000
Lichen_MAGs_cladonia3	*Sphingomonadaceae*	70.6	0.4	2,507,990	68.6	Medium	CAHJWP000000000
Lichen_MAGs_cladonia4	*Burkholderiaceae*	86.0	2.1	5,800,883	60.4	Medium	CAHJWQ000000000
Lichen_MAGs_cladonia5	*Rhodospirillales*	72.9	4.7	4,389,355	65.7	Medium	CAHJWS000000000
Lichen_MAGs_cladonia6	*Acetobacteraceae*	70.4	6.3	4,076,907	68.1	Medium	CAHJXI010000000
Lichen_MAGs_cladonia7	*Acidobacteriaceae*	97.2	6.2	4,643,711	57.8	Medium	CAHJWN000000000
Lichen_MAGs_lobaria1	*Acidobacteriaceae*	98.9	3.6	3,786,442	61.3	High	CAHJWL010000000
Lichen_MAGs_lobaria2	*Myxococcales*	90.8	3.1	5,830,418	63.7	High	CAHJWM010000000
Lichen_MAGs_lobaria3	*Sphingobacteriaceae*	97.0	3.0	3,839,488	39.2	High	CAHJWG010000000
Lichen_MAGs_lobaria4	*Acidobacteriaceae*	96.2	1.7	3,656,062	60.4	High	CAHJWI010000000
Lichen_MAGs_lobaria5	*Verrucomicrobia*	91.3	2.0	4,381,859	63.2	High	CAHJWO010000000
Lichen_MAGs_lobaria6	*Chitinophagaceae*	97.4	2.6	4,287,644	35.8	High	CAHJWK010000000
Lichen_MAGs_lobaria7	*Terrabacteria* group	52.4	0.2	2,887,644	62.1	Medium	CAHJWR010000000
Lichen_MAGs_lobaria8	*Rhizobiales*	76.7	0.9	3,213,075	68.9	Medium	CAHJXE010000000
Lichen_MAGs_lobaria9	*Sphingobacteriaceae*	87.1	1.1	4,667,688	42.1	Medium	CAHJXH010000000
Lichen_MAGs_lobaria10	*Sphingomonas*	77.7	2.4	2,792,940	68.1	Medium	CAHJXA010000000
Lichen_MAGs_lobaria11	*Betaproteobacteria*	87.8	2.2	3,713,330	68.9	Medium	CAHJXF000000000
Lichen_MAGs_lobaria12	*Sphingomonas*	66.5	2.1	2,627,184	69.0	Medium	CAHJWX010000000
Lichen_MAGs_lobaria13	*Sphingomonas*	77.5	1.3	2,846,736	68.8	Medium	CAHJWW010000000
Lichen_MAGs_lobaria14	*Rhodospirillales*	80.7	1.2	3,128,862	67.0	Medium	CAHJWU010000000
Lichen_MAGs_lobaria15	*Sphingomonadaceae*	82.1	0.0	2,472,747	69.6	Medium	CAHJWT010000000
Lichen_MAGs_lobaria16	*Deltaproteobacteria*	26.9	1.5	880,962	69.6	Low	CAHJWZ000000000
Lichen_MAGs_lobaria17	“*Candidatus* Parcubacteria”	40.0	1.1	492,776	48.0	Low	CAHJXB000000000
Lichen_MAGs_peltigera1	*Burkholderiales*	83.8	5.5	4,417,129	63.3	Medium	CAHJWV010000000
Lichen_MAGs_peltigera2	*Rhodospirillales*	63.7	1.0	3,333,202	69.5	Medium	CAHJXG010000000
Lichen_MAGs_peltigera3	*Sphingomonadaceae*	54.1	0.9	1,837,053	68.5	Medium	CAHJXC010000000
Lichen_MAGs_peltigera4	*Sphingomonas*	83.7	1.1	2,414,019	67.0	Medium	CAHJXD010000000
Lichen_MAGs_peltigera5	*Comamonadaceae*	27.5	1.7	1,671,302	65.3	Low	CAHJWY010000000

### Data availability.

This shotgun metagenome project with three lichen metagenomes has been deposited in the European Nucleotide Archive (ENA) database under the study number PRJEB38505 and accession numbers ERR4179389 to ERR4179391 for the data sets. The MAG sequences are accessible under the accession numbers provided in Table 1.
